# The infrapatellar fat pad in inflammaging, knee joint health, and osteoarthritis

**DOI:** 10.1038/s41514-024-00159-z

**Published:** 2024-07-15

**Authors:** Magnolia G. Wang, Patrick Seale, David Furman

**Affiliations:** 1 Department of Biology, School of Arts and Sciences, Philadelphia, PA 19104 USA; 2 Institute for Diabetes, Obesity and Metabolism, Perelman School of Medicine, Philadelphia, PA 19104 USA; 3 Department of Cell and Developmental Biology, Perelman School of Medicine, University of Pennsylvania, Philadelphia, PA 19104 USA; 4 Center for AI and Data Science of Aging, Buck Institute for Research on Aging, Novato, CA 94945 USA; 5 Stanford 1000 Immunomes Project, Stanford University, Stanford, CA 94305 USA; 6 IIMT, Universidad Austral, Consejo Nacional de Investigaciones Científicas y Técnicas, Pilar, 29 Argentina

**Keywords:** Inflammation, Immunological disorders

## Abstract

Osteoarthritis (OA) is the most common form of arthritis and accounts for nearly $140 billion in annual healthcare expenditures only in the United States. Obesity, aging, and joint injury are major risk factors for OA development and progression, but the mechanisms contributing to pathology remain unclear. Emerging evidence suggests that cellular dysregulation and inflammation in joint tissues, including intra-articular adipose tissue depots, may contribute to disease severity. In particular, the infrapatellar fat pad (IFP), located in the knee joint, which provides a protective cushion for joint loading, also secretes multiple endocrine factors and inflammatory cytokines (inflammaging) that can regulate joint physiology and disease. Correlates of cartilage degeneration and OA-associated disease severity include inflammation and fibrosis of IFP in model organisms and human studies. In this article, we discuss recent progress in understanding the roles and regulation of intra-articular fat tissue in regulating joint biology and OA.

## Introduction

With an economic burden of almost $140 billion^1^, osteoarthritis (OA) is the most prevalent joint disease and a major cause of pain and disability in adults, estimated to affect one in every seven persons in the US^2^. OA most commonly affects the knee joints, but also can affect the hand, hip, and spine^3^. OA is characterized by progressive cartilage damage, osteophyte (bone spur) formation, meniscal degeneration, and low-grade synovial inflammation, resulting in deterioration and pain^4^. It can present as a monoarticular disease, but up to 50% of patients have multiple joints involved^5^.

Aging is a major risk factor for OA, and as the world’s population ages, the global disease burden of OA is rising^6^. Approximately 50% of symptomatic knee OA (KOA) patients are diagnosed by age 55, and greater than 75% are diagnosed by age 65^7^. Aging is linked with elevated circulating levels of pro-inflammatory cytokines (inflammaging)^8^, which promote pathology of cartilage, synovium, and bone within the joint^9,10^. This is coupled with the accumulation of senescent cells, which robustly secrete pro-inflammatory and disease-promoting factors (the senescence-associated secretory phenotype, SASP)^11^.

Another risk factor that can severely impact OA pathology is obesity. It is believed that it does so by different mechanisms involving mechanical stress and tissue-derived inflammation. First, increased body weight puts excess stress on weight-bearing joints like the knee, which can lead to reduced mechanical function and injury^12^. Second, obesity can cause or exacerbate systemic inflammation and metabolic complications, promoting OA development^13^. Interestingly, obesity (and its link with aging) is associated with pathologic changes in joint tissues, including enlarged local adipose (fat) depots.

A more systemic effect of obesity in OA development involves the presence of adipose tissue in other parts of the body distant from the affected joint. Adipose tissue is a heterogeneous and plastic organ that plays a central role in regulating systemic metabolism as well as obesity- and aging-related diseases. There are two main types of adipose tissue, white and brown, organized into several discrete depots located throughout the body^14^. White adipose tissue (WAT) functions primarily to store and release energy in response to changes in systemic energy levels. By contrast, brown adipose tissue (BAT) is specialized to expend energy in the form of heat and can thereby protect animals against hypothermia and obesity. Aging and obesity induce the pathologic remodeling and dysfunction of adipose tissue, promoting fibrosis, systemic inflammation, and diminished expression of the insulin-sensitizing hormone adiponectin. Impaired WAT function in aging and obesity is a major driver of insulin resistance and cardiometabolic disease^15^, whereas reductions in BAT capacity can also exacerbate obesity and metabolic sequelae. Local adipose depots in OA joint tissues consist predominantly of lobulated white adipocytes, which are embedded in fat- and mostly collagen-type-I- and type-III-containing connective, fibrous tissue^14,16^.

**Fig. 1:**
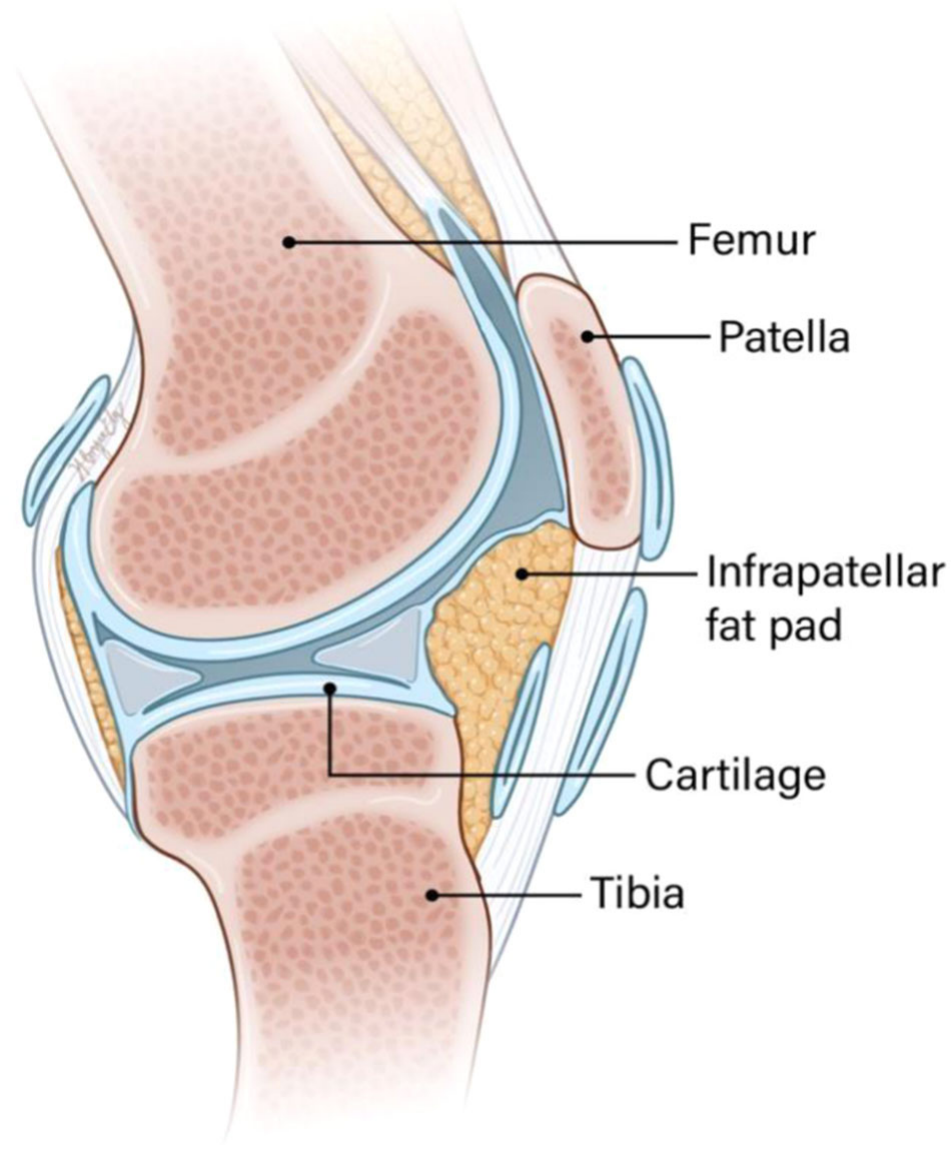
Sagittal view of IFP in the knee joint. The IFP (Hoffa’s fat pad) is positioned at the front of the knee, behind and below the patella, and lined by the synovial membrane on its posterior side facing the joint space. The IFP is arranged into discrete lobules that are surrounded by thin connective tissue, and each lobule consists of unilocular adipocytes that occupy the majority of the volume, along with blood vessels, nerve fibers, and several types of immune cells. The IFP serves to reduce knee load, enlarge synovial space, support patella tendon blood supply, and distribute lubricant in the joint space.

The close connection of adipose tissue with aging- and obesity-related disease points to a critical role of fat tissue in OA development and progression, which is the central topic in this review with a focus on the infrapatellar fat pad (IFP) in the knee. Recent work indicates that IFP interacts with other local tissues to regulate joint homeostasis and pathogenesis, supporting the concept of OA being a “whole joint” disease that affects many tissues including IFP. A better understanding of pathological mechanisms could reveal new treatment avenues (Fig. 1)^17^.

## The Infrapatellar Fat Pad (IFP)

### Anatomy, composition, and physiological function

Fat deposits are present in and around many different joints, including the knee, hip, elbow, and ankle. The knee has several distinct fat pads, including the quadriceps fat pad, pre-femoral fat pad, posterior fat pad, and IFP. The most studied is IFP, also known as Hoffa’s fat pad, situated at the front of the knee, behind the patella and patellar tendon, and adjacent to the menisci (Fig. 1). The IFP is lined by the synovial membrane on its posterior side facing the joint space^18^. Since IFP and synovium are anatomically adjacent and functionally overlapping, a clear distinction between these two types of tissue remains challenging and they are often considered as a single functional unit. The IFP, like other fat depots, is organized into discrete lobules, each filled with unilocular adipocytes that occupy most of the volume and are surrounded by thin connective tissue. The IFP contains blood vessels, nerve fibers, and many immune cell populations.

The IFP serves multiple physiological roles, including reducing the knee load, enlarging the synovial space, supporting patella tendon blood supply, and distributing lubricant in the joint space^19^. Its location allows the adipocytes to act as a cushion by traversing the gaps between joint tissue, functioning like a shock absorber between the anterior tibial plateau and patellar tendon. When the knee joint is in motion, the range of movement within the joint changes the pressure and volume of IFP which helps to limit knee movement and stabilize the patella. An increase in IFP stiffness compared with other fat tissues was reported among patients with OA^19^. In summary, IFP is essential to enable the full extent of motion in the knee joint and protect against joint damage by decreasing biomechanical loading during movement.

The IFP constitutes a small portion of total fat mass and thus does not likely contribute significantly to whole-body metabolic and energy homeostasis. Interestingly, IFP size and lipid storage are preserved under extreme starvation conditions while the main adipose tissue depots export lipids and shrink, suggesting the importance of IFP in maintaining proper knee joint function^20^. Table 1 provides a comprehensive comparison of IFP with the subcutaneous (SAT) and visceral (VAT) adipose tissues.

**Table 1:** IFP in comparison to SAT and VAT

	Infrapatellar Fat Pad (IFP)	Subcutaneous Adipose Tissue (SAT)	Visceral Adipose Tissue (VAT)
Location & Cellular composition	Located in the knee joint, between the joint capsule and synovial membrane; white fat adipose tissue with lobules containing collagenic stroma, fibroblasts, macrophage, and other cell types	Non-ectopic depot organized into upper region and lower region located in the trunk and gluteo-femoral areas respectively, accounts for 80% of body fat	Present in mesentery and omentum, account for 10–20% of body fat; larger size adipocytes and more prevalent infiltrating inflammatory cell types than SAT
Vascularity	Network of arteries abundantly supplied by superior and inferior genicular arteries; irregular connection to the medial genicular artery; multiple anastomoses with vessels of menisci (anterior), patellar tendon (anterior), and tibial periosteum (inferior)	Blood flow and oxygen supply are decreased in obesity; higher capillary density and angiogenic capacity compared to visceral depot; venous drainage through systemic veins	More vascular than SAT, rich in blood supply, visceral fat venous drainage into the portal circulation, with direct hepatic access
Innervation	Nerve fibers predominantly derive from the posterior tibial nerve; free nerve fibers contained within adipose lobules and connective tissue that contain corpuscles along interlobular septa; abundant substance-P nerve fibers	Parasympathetic motor neurons project into subcutaneous compartments; possess vagal input; somatotopic organization attributed to selective innervation by both parasympathetic and sympathetic nervous systems; these autonomic motor centers selectively affect catabolism and/or anabolism	More heavily innervated than SAT, more sensitive to catecholamine-induced lipolysis, and less sensitive to α_2_-adrenergic receptor-dependent inhibition of lipolysis
Physiological function	Acts like a mechanical shock absorber, and facilitates the distribution of synovial fluid to dampen external stress during articular activity	Physiological buffer for additional energy intake during periods of limited energy expenditure, more insulin sensitive; thermoregulatory/thermogenic, prevents heat loss through insulation; protective cushion to external physical stress	Visceral adipocytes are more metabolically active with greater lipolytic activity and a higher rate of insulin-stimulated glucose intake; more insulin-resistant
Role in inflammation	Abundant source of pro-inflammatory adipokines, interleukins, and growth factors that can influence the metabolism of cartilage and synovial membrane in an endocrine-paracrine and autocrine-like manner	Obesity-associated immune cell infiltration interferes with differentiation and/or recruitment of beige adipocytes; an abundant source of adipokines, including leptin, adiponectin	More prevalent infiltrating inflammatory cell types than SAT; major systemic source of adiponectin, angiotensinogen, TNF-a, IL-6, CRP, and plasminogen activator inhibitor-1
Response to fasting and disease association	Resistant to starvation-preserved under extreme starvation conditions; pathology of IFP associated with increased risk of osteoarthritis and joint pain	Dramatic changes in fat mass and morphology of depots in response to long-term fasting; stored triglycerides are readily mobilized; triggers greater sympathetic drive to inguinal white adipose tissue (iWAT) compared to visceral depots	Visceral fat accumulation is associated with hyperglycemia, hyperinsulinemia, hypertriglyceridemia, and increased risk of insulin resistance syndrome, diabetes, cardiovascular disease, hypertension, and stroke

### Aging- and obesity-associated pathology

Aging or obesity generally leads to an increase in IFP size and local chronic inflammation^21–23^. In rats, aging increases IFP secretion of pro-inflammatory cytokines tumor necrosis-alpha (TNF-ɑ) and IL-13, as well as diminishing the activity of resident anti-inflammatory M2 macrophages^24^. Larger IFP volume is positively correlated with clinical symptoms and pain, particularly in the patellofemoral compartment^25,26^. Additionally, IFP size and inflammation are associated with cartilage defects, osteophytes, and bone marrow lesions^27^.

Obesity induces the expansion and pathologic remodeling of IFP, like the effects in other WAT depots. A study comparing IFP and synovium from obese versus lean patients with end-stage OA of the knee found that obese patients had larger (hypertrophic) adipocytes, more inflamed and fibrotic synovium which expressed higher levels of toll-like receptor 4 (TLR4)^28^, an innate immune pattern-recognition receptor whose activation leads to NF-κB mediated intracellular signaling and pro-inflammatory cytokine induction^29^. Mice fed a high-fat diet (HFD) with weight gain also tend to develop OA features, including cartilage loss and the development of osteophytes. In addition, these mice display adipocyte hypertrophy and IFP enlargement, along with elevated production of pro-inflammatory cytokines and adipokines such as visfatin and leptin^30,31^. On the other hand, one study found that mice on long-term HFD display an absence of inflammation, macrophage infiltration, and M1 macrophage polarization in IFP. Magnetic resonance imaging (MRI) and histomorphology revealed increased IFP volumes and fibrosis, but an absence of adipocyte hypertrophy^22^. This finding suggested that long-term HFD may trigger compensatory adipocyte hyperplasia via progenitor cell differentiation in IFP, which mitigates inflammation. A large body of literature suggests that adipocyte hyperplasia is metabolically beneficial and can suppress inflammation^32^. Thus, whether obesity predominantly induces hyperplasia or hypertrophy may be an important determinant of disease progression.

Aging induces the maladaptive accumulation of senescent cells that produce inflammatory cytokines and matrix-degrading enzymes in many organs including IFP^33,34^, such as SASP which is causally linked to many aging-related pathologies^35,36^. Interestingly, inflammatory aging (inflammaging)-related cytokines such as the Monokine-Induced by Interferon (MIG)/CXCL9 chemokine^37^ have been found in the serum of OA patients^38^, which highlights the role of age-related systemic inflammation in the development and progression of the disease. Additional SASP factors including ICAM, IL-1β, MCP4, MIF, MMP13, and RANTES promote an inflammatory state that is strongly associated with the pathogenesis of OA^39^. Mass spectrometry identified such SASP factors in synovial fluid of knee OA patients. Notably, some SASP factors, including Gc-globulin and α1-microglobulin, can signal via TLR4 to drive the synthesis of pro-inflammatory cytokines implicated in OA^40^.

## IFP, inflammation, and OA

A popular model for OA development posits that joint loading and repeated stress on cartilage and other joint tissues stimulates the release of damage-associated molecular patterns (DAMPs) and other inflammatory molecules that trigger joint inflammation through several receptor-mediated pathways^41^. Chondrocytes respond to cartilage injury by upregulating certain inflammatory receptors on their surface, including chemokine receptors and TLRs, which, upon ligand activation can stimulate catabolic enzyme secretion^42,43^. The resulting enzymatic degradation of articular matrix components can liberate additional DAMPs which further activate immune cells including macrophages, B cells, and T cells to perpetuate synovial inflammation and inflammatory tissue damage in the joint. The IFP is a site in which the local inflammatory response can be amplified, especially in older and obese subjects^44^.

Indeed, IFP produces many factors with the potential to affect disease progression through paracrine mechanisms, including cytokines, adipokines, growth factors, and fatty acids and lipid derivatives^45^.

### Fatty acids and derivatives

Adipose tissue is specialized for the storage and release of lipids as energy substrates. Under catabolic states, lipid stores in the major fat depots are hydrolyzed via lipolysis into free fatty acids (FFAs) and glycerol for release into circulation. In addition to serving as an important energy source for cells, FFAs exert potent immune modulatory properties. The pro-inflammatory effects of saturated fatty acids have been well documented in the alteration of cell membrane fluidity, modulating cell surface receptor signaling, and binding to the peroxisome proliferator-activated receptor (PPAR) family of nuclear receptors^46^. Fatty acid derivatives, such as prostaglandins (PGs), leukotrienes, and lipoxins derived from arachidonic acid, also play critical roles in regulating inflammatory responses^47^. Lipidomics analysis of IFP-conditioned medium from OA patients undergoing joint-replacement surgery and healthy individuals found decreased levels of the anti-inflammatory lipid mediator lipoxin A4, as well as increased levels of pro-inflammatory factors thromboxane B2 and arachidonic acid in OA subjects^48^. The IFP of OA patients secreted higher levels of arachidonic fatty acid, which increases PGE2 production and worsens cartilage inflammation. On the other hand, IFP of OA patients also released higher amounts of polyunsaturated docosahexaenoic FA, which lowers the production of proinflammatory cytokines, cartilage-degrading enzymes, and COX-2^49^. Therefore, IFP from OA joints can secrete both disease-aggravating and disease-protective factors. Further understanding of mechanisms regulating the balance between pro-inflammatory and regulatory fatty acids and derivatives within IFP in health and disease is needed.

### Adipokines

Adipokines are soluble molecules secreted by adipocytes that may contribute to joint remodeling and inflammation. Several IFP-secreted adipokines are implicated in OA development, including leptin, resistin, adiponectin, serpin peptidase inhibitor clade E member 2 (SERPINE2 gene), fatty-acid binding protein (FABP4), glycoprotein NMB, WNT1 inducible signaling pathway protein (WISP2), and chemerin (Table 2)^50,51^. Recent studies showed that IFP and synovium from knee OA patients expressed higher levels of leptin, chemerin, and FABP4 compared to healthy control subjects^52,53^. Another study in which IFP from patients with end-stage OA was compared to patients with ACL injury found significantly higher levels of adiponectin, leptin, and FABP4 levels in OA patient samples^54^.

**Table 2:** Summary of IFP-associated Cytokines, Growth Factors, and Adipokines in OA

	Source	Roles in OA Pathogenesis	Association with Clinical Features	Reference
IL-1β	Macrophage	Promote cartilage degeneration; induce IL-6, IL-8 and NGF; contribute to hyperexcitability of pain sensory neurons; modulate CLR expression in synovial cells	Contribute to neurogenic inflammation and cause neuropathic pain	J. Rheumatol. (2008)^102^.
IL-6	CD4( + ) T cell Macrophage	Promote cartilage degeneration; responsible for hypersensitivity and hyperalgesia; mediate cartilage degeneration	Contribute to neurogenic inflammation and cause neuropathic pain	J. Funct. Morphol. Kinesiol. (2017)^103^.
IL-8	Macrophage	Stimulate production of IL-1β and IL-6; induce chemotaxis and neutrophil degranulation	Contribute to neurogenic inflammation and cause neuropathic pain	Sci. Rep. (2021)^104^.
VEGF	Macrophage	Promote angiogenesis process; responsible for MCP-1 and IL-6 infiltration; mediate tissue damage; contribute to synovial proliferation and fibrosis	Promote pain	Sci. Rep. (2017)^105^.
Leptin	Adipocyte	Stimulate production of IL-1β and TGF-β; activate mast cell and Th1 lymphocyte; increase and activate MMPs; stimulate the release of collagen	Knee pain associated with increased level of leptin in SF; promote cartilage degradation	Medicine. (Baltimore, 2018)^106^.
Resistin	Adipocyte	Function as pro-inflammatory cytokine; increase production of IL-6, IL-12, and TNF-α; contribute to proteoglycan loss in cartilage; increase cartilage degradation	Levels correlated with symptoms and severity; levels positively associated with IFP signal intensity alteration	Mediators Inflamm. (2019)^107^.
Adiponectin	Adipocyte	Acts as a pro-inflammatory cytokine in OA; increase MMP and NO production; damage cartilage matrix	Knee pain associated with decreased adiponectin/leptin ratio in SF; plasma levels associated with clinical and imaging severities	Front. Cell. Dev. Biol. (2022)^108^.

OA patients exhibit higher plasma and synovial leptin concentrations than healthy subjects, which has been correlated with the extent of cartilage degradation as measured via histology^55^. Notably, in OA patients synovial leptin concentration is up to 10 times greater than plasma leptin concentrations, suggesting that local leptin may impart a greater influence on OA development^56^. Leptin regulates the synthesis of ALP, TGF-β, osteocalcin, and type I collagen in osteoblasts, but can also act on articular chondrocytes to increase the expression and activity of matrix metalloproteinases (MMPs) and stimulate collagen release^57,58^. In the setting of OA, leptin facilitates the production of NO, PGE2, IL-6, and IL-8 production in human chondrocytes through NF-KB and MAPK/JNK pathways^59^. Leptin also increases IL-1β secretion as well as mast cell and Th1 cell activation. Together, these findings demonstrated that leptin signaling is a complex network involving multiple target cells which may play important roles in cartilage and contribute to clinical symptoms of OA. Of note, a study in leptin-deficient (ob/ob) mice and leptin receptor-deficient (db/db) mice demonstrated extreme obesity and reduced subchondral bone thickness compared to controls, without increased incidence of knee OA or differences in serum inflammatory cytokine levels^60^. These results indicate that adiposity alone in the absence of leptin signaling is not sufficient to promote OA.

Another important cytokine that participates in the pathophysiology of OA is resistin which is believed to trigger macrophages to secrete pro-inflammatory cytokines IL-6, IL-12, and TNF-ɑ, in IFP and synovial fluid^61^. Resistin also promotes proteoglycan loss in cartilage, and its increased levels of resistin correlate with KOA severity and symptoms in patients^62^. Intra-articular injection of resistin triggered degradation of cartilage and subchondral bone, and inflammatory cell filtration of synovia^56^. Although a key pro-inflammatory marker in OA joints, resistin has also been shown to attenuate cytokine production and pro-inflammatory pathways in human dendritic cells, and thus it is important to consider its dual immunomodulatory effects^63^.

The resistin-related adipokine, adiponectin, can promote insulin sensitivity and is generally associated with lower levels of systemic inflammation^64,65^. However, in the joint, adiponectin has been proposed to exert pro-inflammatory effects. Its levels in plasma are positively associated with clinical and imaging severity of KOA^66^. Furthermore, adiponectin elevates MMPs and nitric oxide (NO) production in cultured human osteoarthritic chondrocytes^67^. Similarly, adiponectin treatment of cartilage and chondrocytes from knee joints of OA patients increased NO levels and MMP1, MMP3, and MMP13 expression levels^68^. Interestingly, the adiponectin-induced production of MMPs was markedly suppressed by AMP-activated protein kinase (AMPK) and JNK kinase inhibitors, suggesting that these signaling pathways antagonize adiponectin action.

### IFP-associated macrophages and lymphocytes

In OA, synovitis characterized by variable infiltration of macrophages and lymphocytes is common, and accumulating evidence has demonstrated a similar infiltration in IFP. As IFP is lined by synovial tissue, this is not surprising. Indeed, the two adjacent tissues may form a single functional tissue unit in the joint. Cells expressing both M1- and M2-like phenotypes have been identified in IFP, with M1 macrophages interacting with adipocytes to promote the release of inflammatory mediators^69^, and M2-like CD206+ macrophages inhibiting cartilage degradation in areas surrounding IFP. M2-like macrophages are commonly regarded as profibrotic cells that exert an anti-inflammatory, protective phenotype during wound healing. Studies have shown that significantly more CD206+ macrophages are present in IFP compared with the subcutaneous fat tissue of the same OA patients^70^, indicating that M2 macrophages (CD14 + CD206 + ) could drive IFP fibrosis, a common finding in OA patients and animal models.

T cells and other leukocytes are also present in IFP. In the OA joint, a higher percentage of mast cells and a lower percentage of T cells were detected in IFP, as compared to subcutaneous adipose tissue. Although the proportion of CD4 + T-helper cells was found to be greater than that of CD8 + T-killer cells in IFP of OA patients, CD8 + T cells have been shown to display higher levels of activation, suggesting a role for cytotoxicity in OA^71^. Obesity is known to disrupt the systemic balance between the Th1 and Th2 populations and further influence macrophage phenotypes and activity, however, it remains to be investigated whether this imbalance extends to the T cell and macrophage populations in IFP.

### Contribution of IFP to cytokine and growth factor production in the joint

It is known that cytokines contribute to the development of OA; IL-1β, IL-6, and IL-8 among others have been identified as key mediators. IL-1β has been shown to stimulate IFP production of inflammatory cytokines, adipokines, and growth factors including TNF-α, IL-6, and VEGF^72^. IFP from OA patients expressed markedly higher IL-6 levels than IFP from patients with ACL injury^73^, and released more IL-6 and soluble IL-6 receptors than subcutaneous adipose tissue (SAT)^74^. Of note, studies in obese OA patients suggest that IFP-derived IL-6 contributes to paracrine inflammation and cartilage matrix degradation^75^. Furthermore, IFP has been shown to secrete IL-8, exuding a profibrotic effect on the synovial membrane as well as promoting the further migration of immune cells into IFP, exacerbating the inflammatory state^76^. OA is associated with striking structural changes in IFP, including a marked increase in inflammatory lymphocytic infiltration and vascularization. OA patients exhibit enhanced expression of VEGF in synovial fibroblasts and SF in comparison to healthy subjects; the positive correlation between VEGF levels and increased vascularization in the synovium reveals an underlying interaction between IFP and surrounding synovium^77,78^. Table 2 summarizes the role of several IFP-associated cytokines, growth factors, and adipokines in OA.

## The IFP and implications for OA

### OA pain

Patients with symptomatic OA experience pain which can cause significant physical disability. Although historically OA pain was thought to derive primarily from the loss of cartilage leading to bones rubbing against each other, it is now clear this is an overly simplistic description that does not address the chronic and changing nature of OA pain throughout the disease. Thus, the mechanisms of OA pain generation and persistence are currently an area of intense investigation.

The IFP is likely to be a key contributor to the pain mechanisms in the knee. IFP is a highly innervated tissue that receives its innervation from the tibial nerve, recurrent peroneal nerve, and common peroneal nerve. The abundance and sensitivity of sensory nerve fibers were demonstrated by intra-articular injections of hypertonic saline into the medial IFP of healthy human subjects, which elicited anterior knee pain^79^. In addition, increased IFP signal intensity in MRI studies (denoting inflammation) was positively associated with OA knee pain^80,81^. In a study of 94 patients with KOA, higher pain scores were found to be significantly correlated with increased leptin levels and decreased adiponectin/leptin ratio in SF, linking pain to adipokines^82^. And, in animal models, extensive fibrosis was identified in the parenchymal region in IFP before the onset of persistent pain development^83^, and anti-fibrotic treatments may alleviate pain^84^.

Neuropeptides, namely substance-P (SP) and calcitonin gene-related peptide (CGRP), are present in sensory nerve fibers. SP-positive nerves are widely present in IFP of patients with anterior knee pain and OA^85^. SP can also stimulate macrophages to increase the production of pro-inflammatory cytokines IL-1β, IL-6, and IL-8, actively perpetuating neurogenic inflammation^86^. Further, CGRP was found in the capillaries of IFP from OA patients; its expression has been associated with cyclo-oxygenase-2 (COX-2) which generates prostaglandin mediators of inflammatory pain. CGRP levels were also higher in IFP than in synovial tissue of OA patients^18^, suggesting that IFP may be a major source of CGRP in the OA joint.

### OA fibrosis

Inflammatory hyperplasia and hypertrophy of IFP were initially characterized by fibrosis and calcifications^87^, accompanied by clinical features of knee pain, impaired mobility, and swelling as a result of repeated microtrauma to IFP. Among the various pathological manifestations, fibrosis is one of the most characteristic changes found in OA patients. Histological examination of OA joints demonstrates a greater degree of fibrosis in IFP than in SAT. Furthermore, increases in IFP fibrosis and inflammation due to aging and obesity may disrupt proper biomechanical functions by limiting the ability of the knee joint to absorb mechanical forces during weight-bearing activity. Thus, fibrosis of IFP likely exerts maladaptive effects on the knee joint, promoting joint damage and increasing susceptibility to OA.

Fibrotic changes in IFP following experimental knee injury were found to be strongly associated with persistent pain symptoms. Intra-articular injection of monoiodoacetic acid (MIA) in rats established an acute inflammatory model of fibrosis^84^. Subsequent injection of C-type natriuretic peptide (CNP), an anti-fibrotic agent that inhibits the TGF-β signaling pathway^88^, blocked fibrotic changes in IFP, reduced articular cartilage degeneration, and alleviated persistent pain. The fibrotic lesions in untreated rats displayed new blood vessel formation and invasion of nerve fiber endings. Therefore, inhibition of fibrosis may decrease neovascularization and innervation within IFP and mitigate pain sensitivity and OA pathology^84^.

Interactions between IFP and synovium can modulate fibrosis and joint function. The increased infiltration of immune cells and secretion of pro-inflammatory mediators in IFP, such as IL-6, can modify the phenotype of synovial fibroblasts and promote fibrosis throughout the joint^74^. In line with this, conditioned medium from IFP promoted a pro-fibrotic phenotype in human fibroblast-like synoviocytes (FLS) from KOA subjects. Reciprocally, cytokines secreted from the synovium can induce inflammatory responses in IFP.

### OA progression

**Fig. 2:**
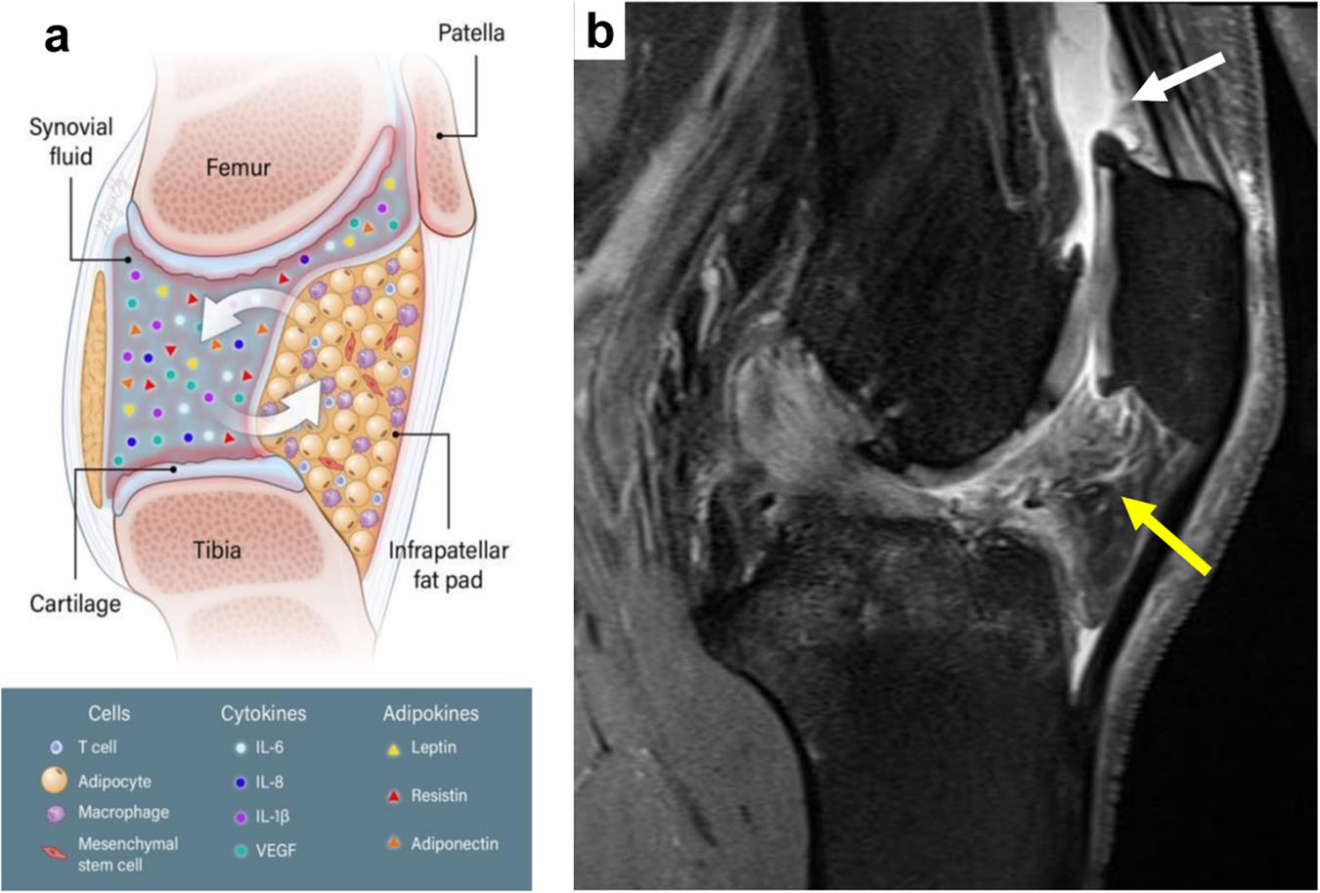
Inflammatory milieu of IFP. **a** Schematic of IFP as both a site and a source of inflammation in the joint, and a contributor to the inflammatory synovial milieu in OA. In addition, IFP can be a rich source of MSCs that can modulate inflammation and potentially play a role in joint tissue repair. **b** Sagittal proton density fat-suppressed Magnetic Resonance image demonstrating altered signal within Hoffa’s (yellow arrow) and the suprapatellar fat pads (white arrow) in a patient with knee OA. (MR image courtesy of Christine Chung, MD, Professor of Radiology, University of California San Diego (UCSD) Department of Radiology.

The properties of IFP as measured by MRI may help predict disease course and progression. MRI studies have shown that OA patients develop larger IFP volumes than healthy subjects, and this volume increases with age. In elderly subjects with OA, the maximum area of IFP was significantly associated with joint space narrowing, medial osteophytes, cartilage volume, and bone marrow lesions (BMLs). In a two-year longitudinal study among elderly subjects who had not yet developed radiographic KOA at the time of enrollment, changes in IFP signal intensity were analyzed along with knee joint structure change over time^89^. Individuals who developed KOA were more likely to display IFP signal intensity alteration than those who did not develop KOA (Fig. 2b). Importantly, the study suggested that adults with IFP signal intensity alteration in the absence of radiographic KOA were at high risk for accelerated disease course, likely as a result of local inflammation. Further, the maximum area of IFP corresponded to yearly reductions in tibial cartilage thickness in elderly women^90^. These results suggested that IFP has significant predictive potential for the development and progression of OA cartilage damage.

### The IFP and future OA therapy

Multiple studies suggest that IFP can be a source of reparative cells for tissue engineering. Mesenchymal stem cells (MSCs) are multipotent cells that can be isolated from adult human tissues including adipose tissue, umbilical cord blood, and bone marrow^91^. MSCs possess immunomodulatory, anti-fibrotic, and anti-apoptotic properties and have been utilized clinically as a source of chondroprogenitor cells for tissue regeneration^92^. MSCs derived from AT may be particularly effective in treating OA-associated inflammation, as shown in animal models and clinical trials^93,94^. These adipose tissue-derived MSCs may have significant chondrogenic potential compared to MSCs from other tissues^95^. Quantitative characterization of progenitor cells from IFP of OA patients aged 50 or older exhibited increased prevalence, concentration, and proliferation potential compared to synovium- or periosteum-derived cells^96^. In addition, MSCs derived from IFP are a preferred tissue source over MSCs from other connective tissue for use in cartilage repair. Stem cells from IFP demonstrated the capacity to generate cartilaginous grafts with a structural and spatial matrix composition more akin to that of native articular cartilage^97^. These tissue constructs are being investigated to repair cartilage defects from joint injuries, and potentially regenerate previously degraded tissues in the context of OA^98^.

MSCs also play critical immunomodulatory roles, which could be harnessed for cell-based therapies. IFP-MSCs demonstrated potent immunomodulation of the activity of synoviocytes and macrophages, which showed significantly reduced cell proliferation, altered inflammatory profiles, and reduced secretion of pro-inflammatory molecules^99^. MSCs derived from IFP of rheumatoid arthritis patients were shown to suppress the proliferation of activated PBMCs, induce IL-10 secretion, and generate FoxP3^+^ Treg cells^100^. Given these features and the favorable location within the joint, IFP represents a promising source of MSCs for cartilage repair and immunomodulation, with the potential to transform future arthritis treatment. Furthermore, recent studies have suggested the use of glucagon-like peptide-1 receptor agonists as a potential therapy for the treatment of OA-related symptoms, as they exhibit immunomodulatory and anti-inflammatory effects^101^.

## Discussion

OA is a painful, multifaceted, and disabling disease driven by the converging influence of aging, obesity, aberrant mechanics, and inflammation. The IFP, located in the knee, has unique features compared to other adipose tissues in the body and has been shown to play multiple important roles in regulating joint function. Cushioning and lubricating were previously considered the only functions of IFP, and recent studies identified this tissue as a critical local modulator of inflammatory and fibrotic responses (Fig. 2a). However, the relative contributions of local versus systemic effects of adipose tissue in the context of obesity and aging remain to be clarified, and future studies isolating the local effects of IFP on OA disease features are warranted.

Recent progress in the field provides optimism for the development of new treatment strategies. In particular, early modulation of inflammation in IFP and synovial membrane may present a promising disease-modifying strategy. In addition, it is also attractive to harness the reparative and immunomodulatory functions of MSCs that reside in IFP. Studies focused on the complex interplay between intra-articular adipose tissue and other joint cell types will continue to provide new insights and may reveal potential therapeutic targets.

## Data Availability

Data sharing is not applicable to this article as no datasets were generated or analyzed during the current study.
